# Cdc45 Limits Replicon Usage from a Low Density of preRCs in Mammalian Cells

**DOI:** 10.1371/journal.pone.0017533

**Published:** 2011-03-01

**Authors:** Philip G. Wong, Sherry L. Winter, Elena Zaika, Thinh V. Cao, Umut Oguz, John M. Koomen, Joyce L. Hamlin, Mark G. Alexandrow

**Affiliations:** 1 Program in Molecular Oncology, Moffitt Cancer Center and Research Institute, Tampa, Florida, United States of America; 2 Proteomics Facility, Moffitt Cancer Center and Research Institute, Tampa, Florida, United States of America; 3 Biochemistry and Molecular Genetics, University of Virginia, Charlottesville, Virginia, United States of America; University of Minnesota, United States of America

## Abstract

Little is known about mammalian preRC stoichiometry, the number of preRCs on chromosomes, and how this relates to replicon size and usage. We show here that, on average, each 100-kb of the mammalian genome contains a preRC composed of approximately one ORC hexamer, 4–5 MCM hexamers, and 2 Cdc6. Relative to these subunits, ∼0.35 total molecules of the pre-Initiation Complex factor Cdc45 are present. Thus, based on ORC availability, somatic cells contain ∼70,000 preRCs of this average total stoichiometry, although subunits may not be juxtaposed with each other. Except for ORC, the chromatin-bound complement of preRC subunits is even lower. Cdc45 is present at very low levels relative to the preRC subunits, but is highly stable, and the same limited number of stable Cdc45 molecules are present from the beginning of S-phase to its completion. Efforts to artificially increase Cdc45 levels through ectopic expression block cell growth. However, microinjection of excess purified Cdc45 into S-phase nuclei activates additional replication foci by three-fold, indicating that Cdc45 functions to activate dormant preRCs and is rate-limiting for somatic replicon usage. Paradoxically, although Cdc45 colocalizes *in viv*o with some MCM sites and is rate-limiting for DNA replication to occur, neither Cdc45 nor MCMs colocalize with active replication sites. Embryonic metazoan chromatin consists of small replicons that are used efficiently via an excess of preRC subunits. In contrast, somatic mammalian cells contain a low density of preRCs, each containing only a few MCMs that compete for limiting amounts of Cdc45. This provides a molecular explanation why, relative to embryonic replicon dynamics, somatic replicons are, on average, larger and origin efficiency tends to be lower. The stable, continuous, and rate-limiting nature of Cdc45 suggests that Cdc45 contributes to the staggering of replicon usage throughout S-phase, and that replicon activation requires reutilization of existing Cdc45 during S-phase.

## Introduction

DNA replication initiates from chromatin-bound pre-Replication Complexes (preRCs). The Origin Recognition Complex (ORC) nucleates preRC assembly on DNA [1], thus identifying the target sequence for initiation. ORC recruits Cdt1 and Cdc6 [2], [3], [4], which facilitate loading of the hexameric Mini-Chromosome Maintenance (MCM) complex [5], [6]. Cdc45, Mcm10, and DNA polymerases are recruited [7], [8], and DNA unwinding occurs in a Cdc45- and MCM-dependent manner [9]. In yeast, Cdc45 and MCMs are also present at elongating forks [10] and required for fork progression [11], [12].

*Xenopus laevis* embryonic extracts replicate sperm chromatin very efficiently, but in a sequence-independent manner from origins that are spaced ∼10–15-kb apart [13], [14], [15], [16]. The distance between these origins defines the replicon size, and these replicons initiate in clusters in a staggered manner [13]. Each replicon/origin in the *Xenopus* system contains approximately one ORC hexamer, two Cdc6, two Mcm10, 1–2 Cdc45, and 20–50 MCM hexamers bound to chromatin prior to S-phase [7], [13], [15], [17], [18], [19]. Thus, each embryonic replicon has one preRC of the above stoichiometry from which to initiate DNA replication. Evidence suggests that Cdc45 binds and selects two of the many available MCM hexamers within each preRC for functioning during unwinding, and Cdc45 is rate-limiting for DNA replication in embryonic extracts [17]. This excess of MCMs for Cdc45 recruitment, along with frequent (10–15-kb) spacing of preRCs, likely contributes to the high efficiency of DNA replication in the embryonic system. Further, embryonic chromatin has dormant origins, via the excess MCMs, whose functionality are important during replicative stress [20].

In a study that mimicked passage of *Xenopus* embryonic extracts through the mid-blastula transition toward a somatic state, replicon size on *Xenopus* chromatin increased eight fold, which was postulated to result from a limiting concentration of a key replication factor [15]. However, the preRC components analyzed were in excess in the extracts [15] and the identity of a rate-limiting factor(s) affecting replicon size remained elusive. Interestingly, one candidate for such a limiting factor that was not analyzed in this prior study is Cdc45. However, a later study indicated that Cdc45 is indeed rate-limiting for embryonic extract DNA replication, but is present in excess in the extracts, suggesting that it is not rate-limiting due to stoichiometric issues [17]. Related to this, in *S. cerevisiae* yeast Cdc45 appears to be present at similar stoichiometric ratios to MCM and ORC subunits [21], but has been found to be rate-limiting for DNA replication *S. pombe* yeast [22].

Mammalian somatic cells have very large replicons that vary from ∼50-kb to 500-kb [23], [24], [25], [26], [27], [28]. As in embryonic extracts, mammalian replicons appear to replicate in clusters and in a staggered manner [23], [25]. However, mammalian origins are far less efficient at firing relative to embryonic origins [27], [29]. We predicted that the biological characteristics of mammalian somatic replicon size, usage, and origin efficiency might be derived at the molecular level from unique characteristics of preRC distribution or subunit availability that differs from embryonic extracts. Toward this end, we used a systematic and overlapping approach to determine the approximate stoichiometry and average genomic distribution of mammalian preRCs. Our results show that significant differences indeed exist between somatic cells and embryonic systems. Mammalian ORC and MCM hexamers are much less abundant in somatic cells, and Cdc45 protein levels are extremely low and limiting for replicon usage in mammalian cells. These results provide a molecular explanation for several biological characteristics of DNA replication in mammalian cells, including why somatic replicon sizes are large, origin efficiency is low, and replicon usage is staggered.

## Results

### Quantification method and antibody validity

The method behind our approach has been used effectively to measure similar protein stoichiometries in yeast and *Xenopus* [5], [7], [17], [18], [19], [30], [31]. In this method, the immunoblotting signal of the protein of interest (but unknown molecular amount) derived from a known number of cells is compared to purified known amounts of the same protein (the standard). Several issues were considered to mitigate potential difficulties in interpretations. Purified standards were used such that the same species of preRC subunit was compared to itself, but also compared across species. Because post-translational modifications to proteins can alter antibody recognition, we performed a heavily redundant analysis to prevent erroneous conclusions that can be drawn from single assays. When quantifying a preRC subunit in most cases, two or more independent antibodies and two or more independent assays on separate lysates were used (and averaged). Also, assays were performed on multiple members of ORC and MCM hexamers under the assumption that the relative stoichiometries obtained would be 1∶1 within each complex if present in hexamers (which proved to be true). Antibodies (Table S1) were verified for specificity on immunoblots (Fig. S1; note this figure is not for quantitative comparisons). This overlapping and redundant approach with multiple antibodies and immunoblots, against multiple subunits predicted to be hexameric, generated a high degree of confidence in the final measurements.

### ORC subunit measurements

**Figure 1:**
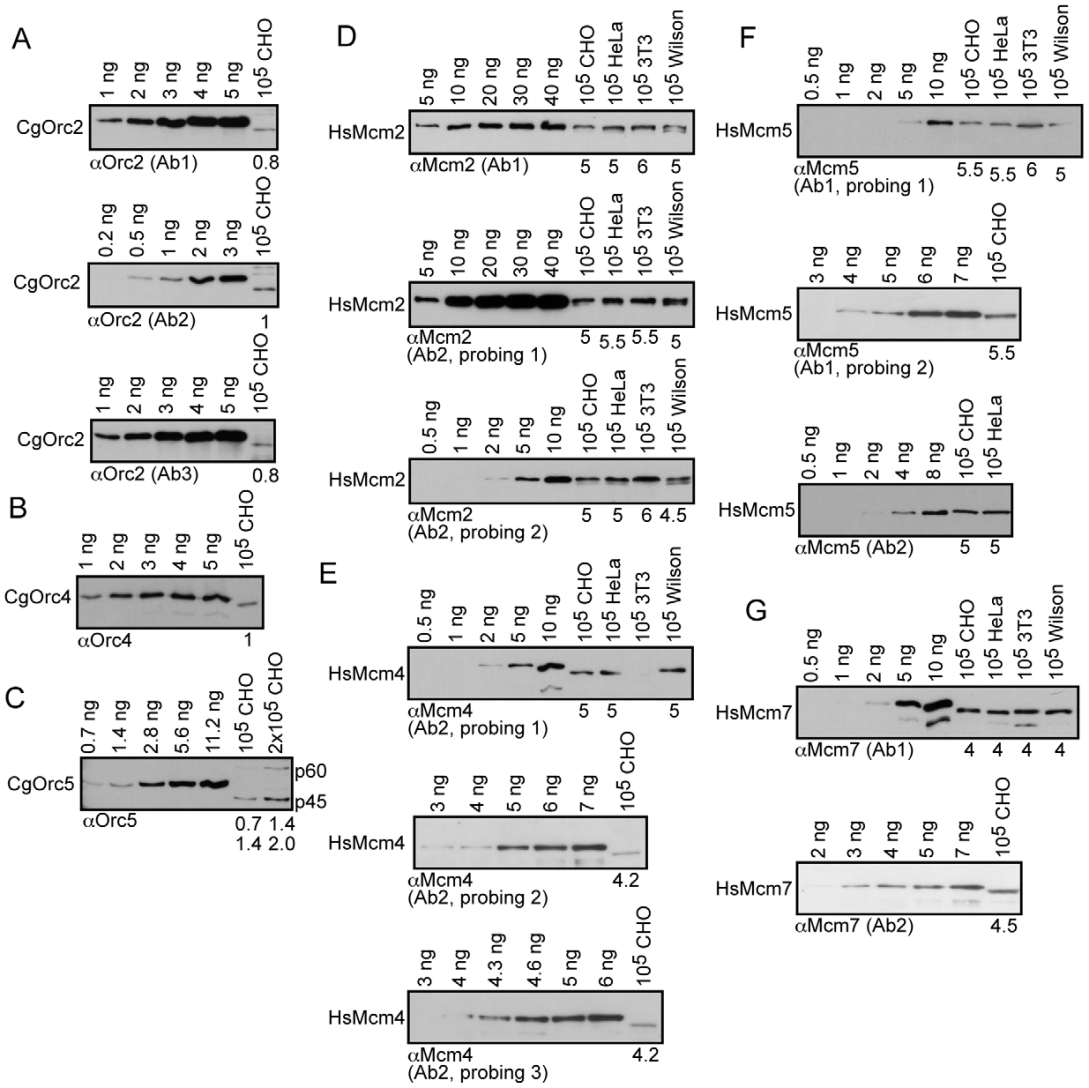
Estimates of ORC and MCM subunit levels in somatic cells. Increasing amounts of bacterially-expressed and purified CgOrc2 (*A*), CgOrc4 (*B*), and CgOrc5 (*C*) Mcm2 (*D*), Mcm4 (*E*), Mcm5 (*F*) and Mcm7 (*G*) were used to estimate the total number of nanograms (ng) of each ORC subunit in the indicated number of asynchronous mammalian cells. Nanograms estimated are shown below each panel and refer to each protein band present (top to bottom). Note that the His6-tags cause standards to migrate slower in gels relative to comparison proteins in lysates.

Whole-cell lysates from asynchronous CHO cells were compared to purified CgOrc2, CgOrc4, or CgOrc5 in duplicate immunoblots performed on separate CHO lysates. Representative results with the ∼ng quantities detected for each subunit are shown in Figure 1A–C, with numerical results in Table S2. The average total cellular level for CgOrc2 obtained with three antibodies is 0.8, 1.0, and 0.8 ng per 10^5^ CHO cells, while CgOrc4 is 1.2 ng/10^5^ cells. p60-Orc5 and p45-Orc5 are 0.7 and 1.2 ng/10^5^ cells, respectively. The p45-Orc5 has been shown by others to be an isoform of Orc5, called Orc5T (for truncated), produced from alternative mRNA splicing [32]. When ORC subunits/cell are calculated, there are ∼7.5×10^4^ CgOrc2, ∼14×10^4^ CgOrc4, ∼7×10^4^ p60-CgOrc5, and ∼16×10^4^ p45-CgOrc5 in each cell. In terms of molecules per genomic region, there are approximately 1 CgOrc2, 2 CgOrc4, 1 CgOrc5 (p60), and 2–3 CgOrc5 (p45) per 100-kb. Although slightly discordant from the 1∶1 ratio expected for subunits in a hexamer, when the chromatin-bound complement of each ORC subunit is considered, these ratios do approach unity (see below).

On average, 1 ORC hexamer exists per 100-kb in somatic cells, which is significantly lower than on embryonic chromatin where 8–10 ORCs exist per 100-kb [13], [15]. Based on such precedents, we had anticipated that there would be more ORC in somatic cells, which is why some of our quantitative blots contain standards loaded at higher levels relative to the unknown lysates (for example, see Fig. 1A, top and bottom anti-Orc2 assays, and the Mcm2 analysis in Fig. 1D, middle panel, discussed later). Such analyses place the comparisons slightly outside of the linear range in those cases, but have been intentionally left in the figures to illustrate that, had there been more ORC, it would clearly have been evident.

We also analyzed lysates from human and murine cells (Fig. S2A). HsOrc4 and MmOrc4, and HsOrc5, produced similar band intensities to CgOrc4/5. Although MmOrc2 showed results similar to CgOrc2, HsOrc2 in HeLa and Wilson cells with two anti-Orc2 antibodies gave very strong signals relative to CgOrc2. Our strain of HeLa cells also contains two forms of Orc2 (Fig. S2A), possibly due to genomic rearrangements, alternative splicing, or post-translational modifications. Although the comparison between CgOrc2 standard and HsOrc2 may give erroneous results due to species differences, it is possible that HsOrc2 is over-represented up to ten-fold in some cells. Indeed, this might explain why 10 fold reduction of HsOrc2 allows viability [33]. In the end, we conclude that MmOrc2 and MmOrc4, and HsOrc4 and HsOrc5, are present at similar levels to that in CHO cells.

### MCM subunit measurements

We possessed purified full-length HsMcm2, HsMcm4, HsMcm5, and HsMcm7. Species-specific comparisons dictated that we analyze human cells, but we also analyzed CHO and 3T3 lysates. In order to mitigate potential problems with comparisons between HsMCM standards and CgMCM subunits, we used a redundant approach involving eight anti-MCM antibodies against four MCM subunits, used on 14 immunoblots, to determine the levels of MCMs per CHO cell. Further, we have cloned and sequenced CgMcm6 and found it to be 96% identical at the amino acid level with HsMcm6, confirming that mammalian MCMs are highly conserved across species. Thus, the anti-MCM antibodies used for these analyses need not have been generated against the cognate mammalian species for obtaining useful quantitation data.

Lysates from asynchronous cells were analyzed for MCM levels (Figure 1D–G). The average total amount for each MCM subunit ranged from ∼4–5.5 ng per 10^5^ human cells. Signal intensities for MCM subunits in CHO and 3T3 lysates were nearly identical to those obtained with human cells in almost all of the immunoblots, indicating that all three mammalian types contain very similar quantities of MCM subunits. Each MCM subunit is present at ∼3–4×10^5^ molecules per somatic cell, producing, on average, ∼4–5 total molecules/100-kb of each MCM subunit (Table S2). These MCM measurements indicate that mammalian cells contain an ∼1∶1 ratio of each MCM subunit, consistent with the complex being a hexamer of equal subunit representation. Further, somatic cells contain significantly fewer MCM hexamers on a per genomic region basis (∼4–5/100-kb) compared to embryonic chromatin (∼200–500/100-kb). This equates to 40-100 fold fewer MCM hexamers available *in total* in somatic cells. Consistent with our results, Knippers and colleagues [34] determined (but did not show the data) that ∼10^6^ HsMcm3 molecules are present per HeLa cell, which is only 2–3 fold above our estimations for Mcm2, 4, 5, and 7, but still vastly lower than on embryonic chromatin.

### Cdc6 and Cdc45 measurements

**Figure 2:**
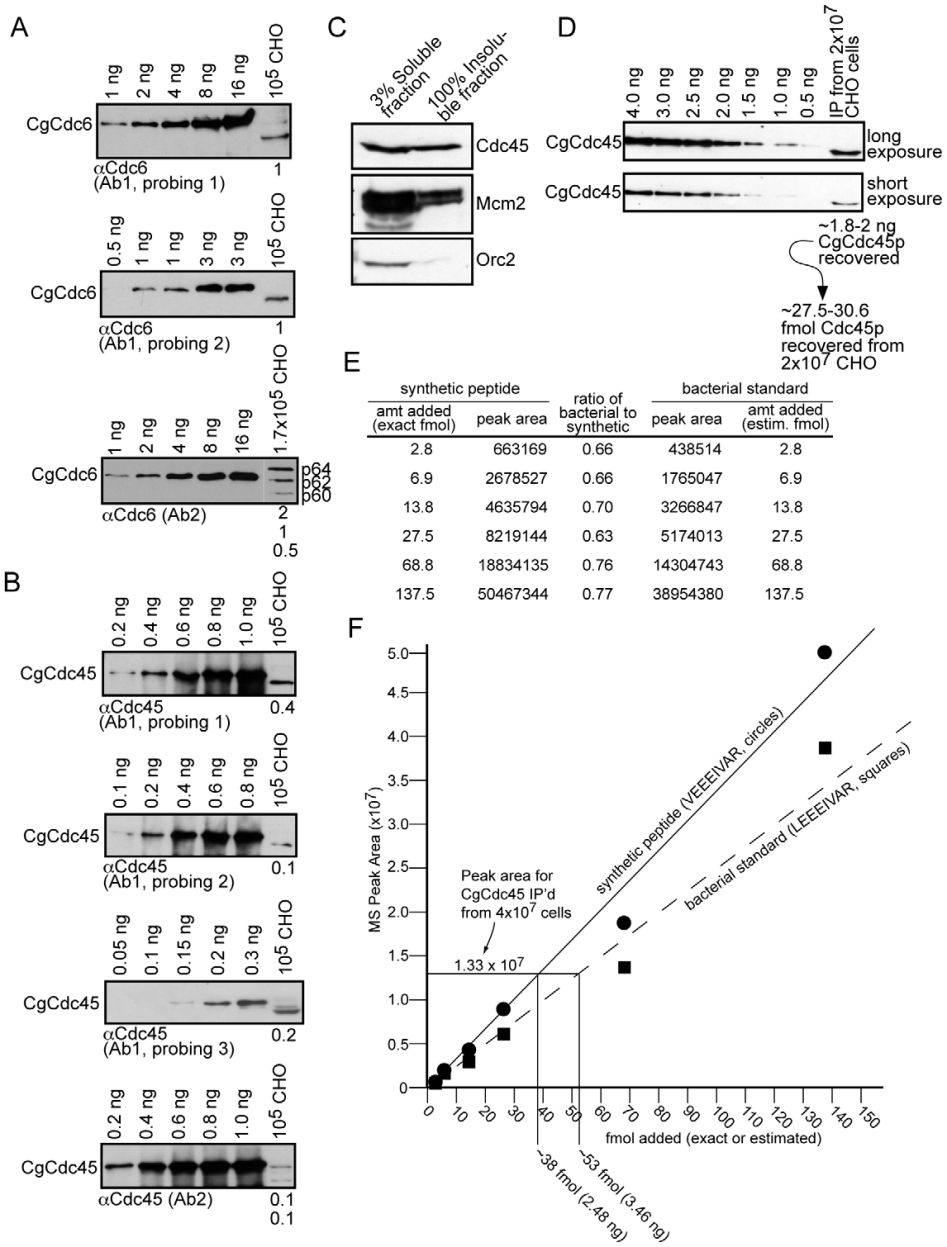
Estimates of Cdc6 and Cdc45 protein levels. Increasing amounts of purified CgCdc6 (*A*) or CgCdc45 (*B*) were used to estimate the total number of nanograms of Cdc6 or Cdc45 in asynchronous CHO cells. Non-tagged CgCdc6 was used for the Cdc6-Ab1 (probing 2) and Cdc6-Ab2 analyses. (*C*) CHO cells were lysed in loading dye, boiled 10 min, sonicated, and spun at high speed for 30 min. The entire insoluble pellet was loaded alongside 3% of the soluble lysate and analyzed by immunoblotting. (*D*) CgCdc45 was immunoprecipitated from 2×10^7^ log CHO cells with chicken anti-Cdc45. The entire immunoprecipitate was blotted against bacterially-generated CgCdc45 standards with rabbit anti-Cdc45. (*E*) QMS analysis was performed on co-analyzed synthetic CgCdc45 peptide and bacterially-generated CgCdc45. (*F*) Standard curves were plotted using the results from *E*, and the CgCdc45 immunoprecipitated from 4×10^7^ CHO cells was compared after QMS.

Several protein bands for Cdc6 and Cdc45 were recognized, which likely represent post-translationally modified proteins. Although mammalian Cdc6 is known to be modified by phosphorylation and ubiquitinylation [35], [36], and at least two distinct protein bands have been shown to exist for mammalian Cdc45 [37], we cannot be certain that all bands recognized are indeed Cdc6 and Cdc45. However, for the purposes of simplifying the quantitation and erring our estimates upward, we determined the total amount of Cdc6 and Cdc45 based on the sum of visible bands detected (and indicate the amounts of individual bands recognized). As such, our quantitation for Cdc45 and Cdc6 must be considered the upper estimates of availability for each protein within the cells. Representative blots with the ∼ng quantities detected are shown in Figure 2A&B, with numerical results in Table S2. 10^5^ CHO cells contain ∼1.15 ng in total of Cdc6 protein, which equates to ∼2 molecules/100-kb, while 10^5^ cells contain ∼0.26 ng of total Cdc45 protein, which represents only ∼0.35 molecules of Cdc45/100-kb. Signal intensities for Cdc6 and Cdc45 in human and murine lysates were very similar to those from CHO (Fig. S2B&C), strongly suggesting that all three mammalian cell types contain similar quantities of Cdc6 or Cdc45. Again, if one excludes the observed additional bands for Cdc6 or Cdc45, and relies only on a single band representing the predicted molecular weight for each protein (*e.g.*, p60-Cdc6, but not p62/p64), then a slightly lower estimate would be obtained for each protein. However, taken as a sum representing the upper estimate, mammalian cells contain ∼2 total Cdc6 molecules relative to each ORC hexamer/100-kb, which is similar to that determined in *Xenopus* extracts (but in the latter Cdc6 exists at ∼2 molecules/ORC/∼10-kb). In contrast, and unlike the embryonic situation, somatic cells have very low levels of Cdc45 even though this is an upper estimate. This is in agreement with another study that found Cdc45 to reside at low levels in human tumor cells [38].

The low levels of Cdc45 were intriguing, and we were concerned that we might be losing Cdc45 protein during the preparative steps, leading to erroneous underestimations. Such loss might be due to insolubility after lysis, poor gel separation, or over-transfer. These potential problems might also exist for ORC and MCM estimations. To verify this was not the case, we pelleted any insoluble cellular proteins using high speed centrifugation, and analyzed protein distribution between soluble and insoluble fractions. The insoluble pellet, although not visible, was loaded in its entirety next to 3% of soluble cell lysate (Fig. 2C). The upper spacer gel and lower resolving gel were blotted against Cdc45, Orc2, and Mcm2. No protein was detected in the upper gel region or on a second membrane behind the primary membrane after doubling transfer duration (data not shown). We did find that ∼3% of Cdc45, Orc2, or Mcm2 was insoluble (Fig. 2C), but could be efficiently separated and analyzed. Thus, we are not excluding proteins in our analytical experiments.

We also used an alternative means to verify our Cdc45 measurements in which we immunoprecipitated Cdc45, estimated the amount immunoprecipitated using immunoblots, and subjected the immunoprecipitated Cdc45 to quantitative mass spectrometric (QMS) verification. Figure 2D indicates that, compared to bacterially-generated Cdc45 standards (bac-CgCdc45, amounts determined against BSA standards), we can recover ∼1.8–2.0 ng of Cdc45 in the immunoprecipitate (IP) from 2×10^7^ CHO cells. This equates to ∼27.5–30.6 fmol of Cdc45 recovered (1 ng = 15.3 fmol). We note that our IP is inefficient and fails to IP >95% of total Cdc45. Two standard curves were generated for QMS analysis of the IP sample, one using a synthetic CgCdc45 peptide of exact amounts, and the other using bac-CgCdc45 that was gel-purified and trypsin-digested. The CgCdc45 tryptic peptide, aa260-LEEEIVAR, was followed due to its high signal intensity in pilot MS assays. The synthetic peptide had a leucine-valine change to offset mass, allowing simultaneous determinations in single QMS runs. QMS comparison of several amounts of bac-Cdc45 to co-analyzed synthetic peptide of the same amounts resulted in peak areas that were, on average, ∼70% of the synthetic peptide signal (Fig. 2E; results are plotted in Fig. 2F). As some loss of protein occurs during tryptic digestion and gel-extraction (but not for synthetic peptide), these results indicate that our BSA-estimated amounts of bac-CgCdc45 are fairly accurate (for this QMS and previous immunoblotting analyses).

To facilitate QMS sensitivity, two immunoprecipitates each from 2×10^7^ CHO were combined for QMS analysis of immunoprecipitated Cdc45 against the standard curves. Fig. 2D predicted that the combined IP would result in a QMS determination of ∼55–60 fmol of Cdc45. As shown in Fig. 2F, the IP sample produced a peak area of ∼1.3×10^7^, calculated to be 38 or 53 fmol, depending on the standard curve that was compared. Although both numbers are slightly lower than predicted, this objective analysis strongly suggests that the immunoblotting determinations for CgCdc45 are accurate estimates (and that we may be over-estimating Cdc45 levels). We conclude that mammalian cells do indeed contain very low levels of Cdc45.

### Chromatin-bound fractions of preRCs and Cdc45 during G1 and S-phase

**Figure 3:**
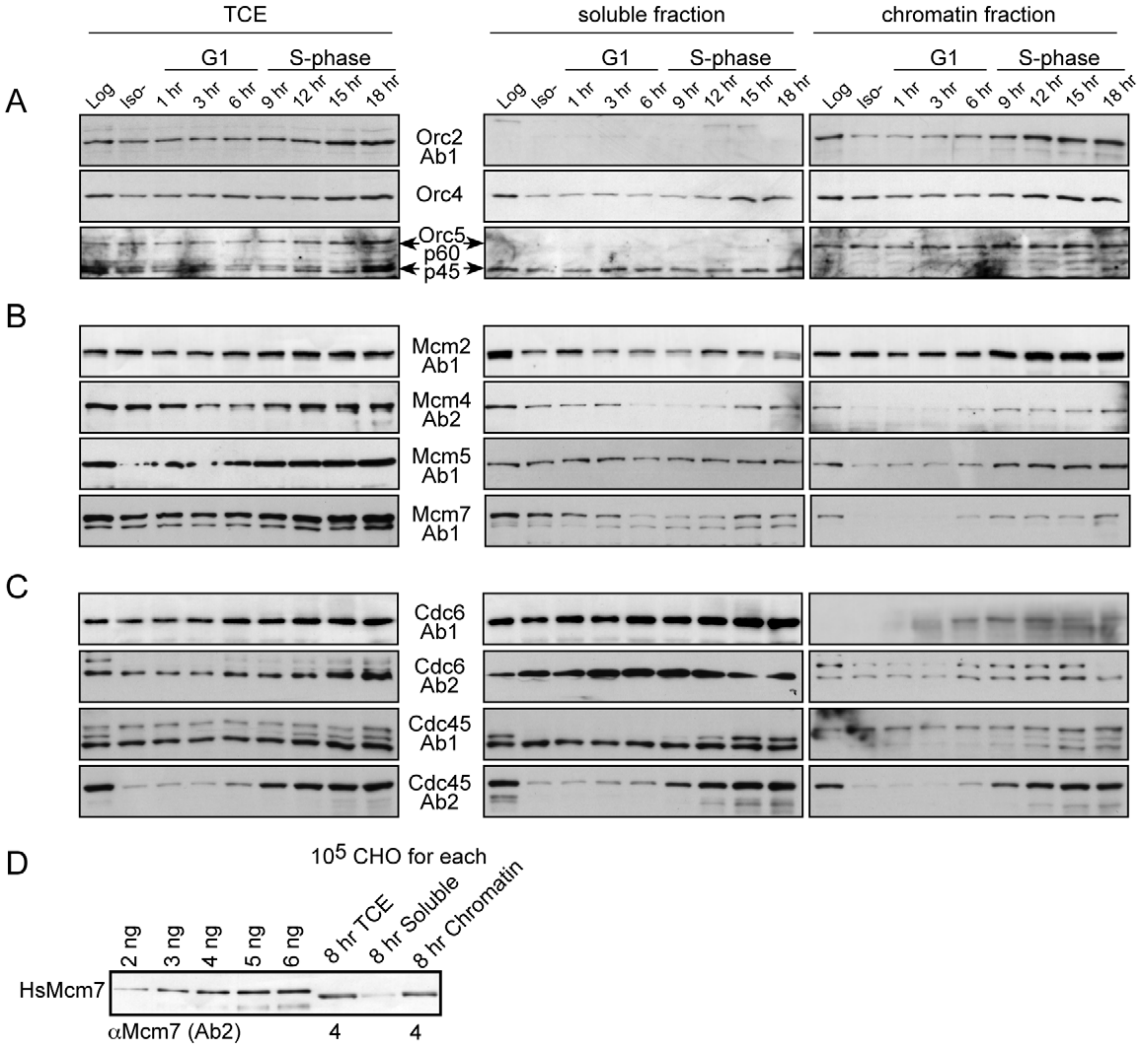
Cell cycle and chromatin distributions of preRC subunits. (*A*–*C*) G_0_-arrested CHO cells were released into G_1_, and samples were collected at the indicated times after release. Total cell extracts (*TCE*) were compared to cytosolic/nucleosolic (*soluble*) and chromatin-bound (*chromatin*) extracts by immunoblotting. Asynchronous (*Log*) cultures were processed identically. Extract from an equivalent amount of cells was loaded in each case, and analyzed with the indicated antibodies. BrdU labeling was ∼50% at 9 hr (G_1_-S transition), ∼95% at 12 and 15 hrs, and ∼80% at 18 hrs (data not shown). (*D*) Quantitation of Mcm7 levels on total and soluble/chromatin fractions in synchronized CHO cells 8 hrs post release.

The molecular estimates described in the previous sections were arrived at using total protein lysates of asynchronous cells. If the subunits are not expressed similarly at all cell cycle stages, then we might be underestimating protein levels in asynchronous cells. Also, it is generally assumed that the chromatin-bound fraction of preRCs are the functional components. To address these issues, we determined the total preRC subunit expression levels during G1 and S-phase, and assessed the chromatin-bound distribution of preRCs at the G1/S transition. Chromatin-bound preRCs are operationally defined by their resistance to extraction with non-ionic detergents [36], [39], which we will refer to as higher affinity, while those extractable we will refer to as lower affinity (often called ‘soluble’ in other reports). Total extracts (*TCE*), lower affinity (soluble), and higher affinity (chromatin) fractions were prepared from equal numbers of synchronized or log CHO cells (Fig. 3). Immunoblotting against lamin and tubulin verified effective fractionation, and BrdU pulsing indicated that G1/S was at 9 hrs and S-phase occurred from 9–18 hrs (data not shown, but see ref. [39]). While G2 and M-phase populations are not represented here, CHO cells have a doubling time of ∼20–21 hrs and G2 and M-phase is 2–3 hrs (data not shown). Therefore, our 18 hr analysis covers most of the cycle.

ORC and MCM dynamics during G1 and S-phase show that none of these subunits fluctuate significantly in total expression during this duration (Fig. 3A&B), with the possible exception of a small decrease in Mcm5 in early G1 (Fig. 3B). Consistent with these findings, another study has shown that Orc2, Mcm3, Mcm4, and Mcm5 do not fluctuate during the cell cycle in asynchronous human cells [36]. Cdc6 and Cdc45 levels also do not significantly fluctuate in TCE samples, although some bands for each are more abundant in S-phase (Fig. 3C). The most notable of these is Cdc45 detected with Ab2, which suggests that we might be underestimating Cdc45 levels by a factor of two with this antibody in log cells. However, if we multiply the results in Table S2 for Cdc45-Ab2 to accommodate this (by two), the outcome is still consistent with that using Cdc45-Ab1 (*i.e.*, very low). We conclude that we are not grossly underestimating ORC, MCM, Cdc6, or Cdc45 subunit levels in log cells.

100% of Orc2, ∼50% of Orc4, 100% of p60-Orc5, and none of p45-Orc5 (Orc5T) displays a higher affinity for chromatin at G1/S (Fig. 3A, 9 hrs). The ∼50∶50 Orc4 distribution has also been seen by others [40]. The log samples, on which we performed subunit calculations above, distribute similarly. Importantly, the bands detected in log samples and at G1/S display similar intensities (for TCE or chromatin), indicating that there are similar total and chromatin-bound numbers of ORC subunits/cell in log samples and at G1/S. Considering the chromatin-bound complement, a 1∶1∶1 stoichiometry exists between Orc2, Orc4, and p60-Orc5 (Table S2; p45-Orc5/Orc5T is excluded due to its non-chromatin-binding nature). We conclude that there is an average of ∼1 chromatin-bound ORC hexamer/100-kb at G1/S.

MCM chromatin-binding dynamics demonstrates that each of the subunits distributes ∼50∶50 between soluble and chromatin fractions at G1/S and in S-phase, as do log samples (Fig. 3B). Others have also shown that ∼50% or less of human MCMs are chromatin-bound during S-phase [34], [36]. The band intensities for MCM log and 9–18 hr samples are similar (for TCE and chromatin), indicating that there are similar total and chromatin-bound numbers of MCM subunits/cell in log samples, and from G1/S through S-phase. We also collected synchronized CHO cells at 8 hrs (∼1 hr prior to G1/S) and quantified Mcm7 levels in TCE and chromatin (Fig. 3D). In agreement with our quantitation for Mcm7 in Fig. 1, just prior to G1/S ∼4–5 Mcm7 reside on chromatin/100-kb, but the fractionation just described indicates that this shifts to ∼50∶50 once S-phase starts.

These results suggest that ∼half of MCM hexamers may be functional (or capable of functioning in the future) in DNA replication at any moment in S-phase. The functional role of chromatin-bound MCMs in DNA replication is derived primarily from embryonic and yeast studies, where MCMs that are not functioning in DNA replication (with lower chromatin affinity) are blocked from entering, or exported out of, the nucleus [4], [41], [42], [43], [44]. However, given that mammalian somatic MCMs are nuclear during the entire cell cycle regardless of chromatin affinities [34], [39], [45], [46], [47], [48], the functional distinction between low and high affinity MCMs in somatic cells is currently unknown. As such, we conclude that somatic cells contain a *total* of 4–5 MCM hexamers per ORC/100-kb, and that *perhaps* ∼2–3 hexamers/ORC are chromatin-bound at any moment in S-phase (Table S2, far right columns and gray boxes). Intriguingly, the latter resembles the embryonic system where ∼2 MCM hexamers/ORC are minimally required for uninterrupted DNA replication [18].

Approximately 33% of total protein bands for Cdc6 and ∼33–50% of total bands for Cdc45 (with Ab1 or Ab2, respectively) become bound to chromatin during S-phase, which is very similar to the distributions for log samples (Fig. 3C). Again, our ∼percentages here must be considered upper estimates, since we are including all visible bands of the appropriate size ranges for Cdc6 and Cdc45 and not purposefully drawing any preferences for particular bands observed. The band intensities for Cdc6 and Cdc45 log and 9–18 hr samples are similar in each case, indicating that there are similar total and chromatin-bound protein numbers per cell in log samples and during S-phase. Thus, an average of ∼0.53 Cdc6 and ∼0.13 Cdc45 (as upper estimates) reside on chromatin relative to each ORC hexamer/100-kb (Table S2). However, since little is known regarding the functional distinctions between low and high affinity forms, it is perhaps more relevant to focus on the total levels for both proteins. Indeed, the chromatin-bound approximations for Cdc6 may be an underestimate of the functional requirement for Cdc6, since in *Xenopus* two Cdc6 are chromatin-bound at each ORC to load MCMs, but can dissociate to load MCMs at other ORCs [18], [49]. As for Cdc45, it exists at an extremely low level genome-wide, whether considering the total amount (∼0.35 Cdc45 per ORC/100-kb, as an upper estimate) or the higher affinity portion (∼0.13 Cdc45 per ORC/100-kb).

### The same limited number of Cdc45 molecules persists through S-phase

**Figure 4:**
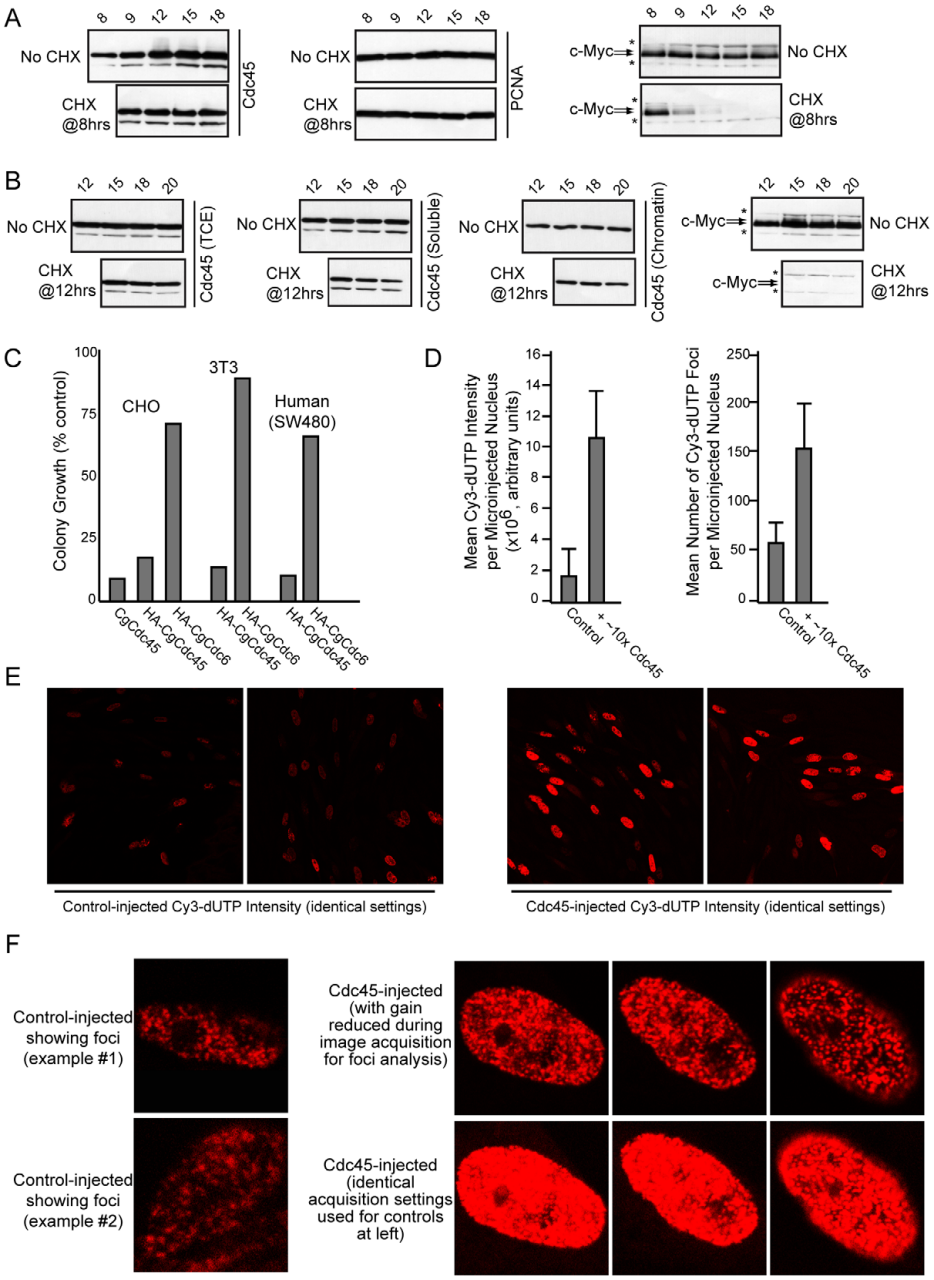
Cdc45 protein is very stable, persists throughout S-phase, and is rate-limiting for replicon usage. (*A&B*) Lysates from synchronized CHO cells analyzed by immunoblotting for Cdc45, PCNA, and c-Myc. Asterisks: background bands. 50 µg/ml cycloheximide (CHX) was added at 8 hrs (lower panels in *A*), or at 12 hrs (lower panels in *B*). BrdU pulsing verified G1/S was at 9 hrs (not shown). For each no-CHX/CHX pair analyzed, data was obtained from a single immunoblot. (*C*) Summary of results for colony forming assays. Data are representative of three assays for each with nearly identical results. (*D*) Graph of results for Cdc45 microinjection experiment. Means of information from 25 most intense, early-S stage nuclei for each condition were plotted +/−1s.d. Raw data is in Table S2. (*E*) Original confocal scans of fields after microinjection, using identical settings for image acquisition. (*F*) Close-up examples of early-S injected nuclei for each condition that were used for foci and intensity computation.

The results of Figure 3C showed that total Cdc45 levels remained largely unchanged during S-phase (9–18 hr). We next determined if this steady-state Cdc45 presence during S-phase was derived from the same limited number of molecules that were continually present throughout the replicative period. Synchronized CHO cells were treated with cycloheximide (CHX) prior to S-phase (Fig. 4A), or within S-phase (Fig. 4B), to block protein synthesis, and the decay of pre-existing Cdc45 was assessed by immunoblotting. In Figure 4B, we also separated lysates into total (TCE), low affinity (soluble), and high affinity (chromatin) in order to analyze different Cdc45 protein bands with varying chromatin affinities. Compared to that seen for the highly labile c-Myc protein, which was acutely degraded in CHX, pre-existing Cdc45 (regardless of chromatin affinity) displayed no lability and remained present for the next 8–10 hrs, the equivalent length of a mammalian S-phase. PCNA was also highly stable. Only a small loss of Cdc45 protein is apparent at 20 hrs, when S-phase is ending (Fig. 4B). The same results were obtained using either anti-Cdc45 antibody (not shown for Ab2), and are in agreement with another study claiming Cdc45 to have a half-life of 10 hrs [38]. Since there is no increase in Cdc45 levels during S-phase (Fig. 3C), and Cdc45 is highly stable for the duration of S-phase, we conclude from these results that mammalian cells have a continual presence of a low number of stable Cdc45 molecules with which they start, progress through, and conclude S-phase.

### Cdc45 is rate-limiting for replicon usage in mammalian cells

Based on studies in other systems, Cdc45 is likely required stoichiometrically at replication forks for MCM function. Further, ∼2 Cdc45 molecules bind MCMs per replicon in embryonic systems [17]. Our results indicate that ∼2 stable Cdc45 molecules exist at any given moment in S-phase in somatic cells for each ∼600-kb of genomic DNA, and the higher affinity Cdc45 is present at ∼2/1400-kb. Since somatic replicons appear to vary from 50- to 450-kb, there is not enough Cdc45 to occupy all replicons simultaneously. This suggests that Cdc45 is limiting for replicon usage in somatic cells, and that the staggering of replicon firing may be attributable in part to the availability of the same limited number of Cdc45 molecules that must be reutilized progressively throughout S-phase for the activation of later-firing replicons.

To test the validity of these predictions, we asked if artificially increasing Cdc45 in mammalian cells altered the dynamics of S-phase and replicon usage. In our first approach, we attempted to stably express exogenous HA-Cdc45 or untagged Cdc45, but found that human, mouse, and hamster cells were significantly reduced in their proliferative capacity when Cdc45 expression was deregulated (Fig. 4C). In contrast, stable expression of Cdc6 did not affect growth. These results suggest that mammalian cells can sense, and do not tolerate, changes to the amount of Cdc45 that is available during the cell cycle. To circumvent this problem, we performed microinjection experiments in which bacterially-generated His6-Cdc45 was placed directly into nuclei already in S-phase, and the effects of the additional Cdc45 molecules on DNA replication and replicon usage were determined. Cy3-dUTP was co-injected and became incorporated into polymerizing DNA during a 30 min period after injection, which served as a direct quantifiable measure of the amount of DNA replication that occurred. We utilized CHO cells in early S-phase to allow us to distinguish and count replication foci produced after injections.

Introduction of ∼10-fold additional Cdc45 produced a significant increase in DNA replication activity, with Cdc45-injected nuclei displaying ∼6-fold the level of replication versus control-injected nuclei (Fig. 4D&E raw data in Table S3). Reducing this to ∼2-fold of additional Cdc45 caused no increase in replication activity (data not shown). The increased replication activity could be due to faster fork movement or additional preRC/replicon activation, or both. Computational determination of the mean number of replication foci produced per nucleus revealed that Cdc45-injected nuclei displayed ∼3-fold the number of replication foci versus controls (Fig. 4D). As fair comparisons, we focused on injected nuclei displaying stage-1 S-phase foci patterns [39] to facilitate such computational analysis (Fig. 4F). This indicated that the additional Cdc45 molecules produced an ∼3-fold increase in the number of preRCs/replicons that were activated during the pulse period after injection. Intriguingly, a 3-fold increase in activated preRCs/replicons would translate to a 6-fold increase in the number of bidirectional forks, consistent with the ∼6-fold increase in mean Cy3-dUTP intensity/nucleus that we observed (Fig. 4D). This suggests that the increased replication/nucleus is due mostly to additional preRC/replicon activation rather than faster fork movement. These results demonstrate that Cdc45 is rate-limiting for DNA replication and replicon usage in somatic cells. Further, given their stable, persistent, and very low levels, Cdc45 molecules need to be reutilized at later-firing replicons to achieve complete replicon usage during S-phase, providing a molecular explanation for why replicon usage is staggered.

### Cdc45 and Mcm2 do not colocalize with active replication sites *in vivo*

Although required for DNA replication to occur, and acting presumably as a helicase, MCMs paradoxically do not colocalize with active replication sites in mammalian cells [50], [51], [52], [53], [54]. Given that Cdc45 is a cofactor for MCM functionality [9], we determined if Cdc45 colocalized with MCM sites and/or active replication foci. Asynchronous CHO cells were pulsed with BrdU to label actively replicating foci in S-phase cells, and cells were fixed and stained by IF for the localization of Mcm2, Cdc45, RPA-32 (single-stranded binding protein, 32 kDa subunit), or DNA polymerase delta. Late S-phase stage nuclei [39] were visualized for clarity of replicating foci colocalization, or lack thereof. For RPA and polymerase analyses, detergent extraction (0.1% TX-100) was used prior to fixation to examine the tightly-bound complements of each factor known to associate with replication foci. For Cdc45 and Mcm2, we performed no detergent extraction in order to visualize the total complement (chromatin-bound and non-bound) of each protein.

**Figure 5:**
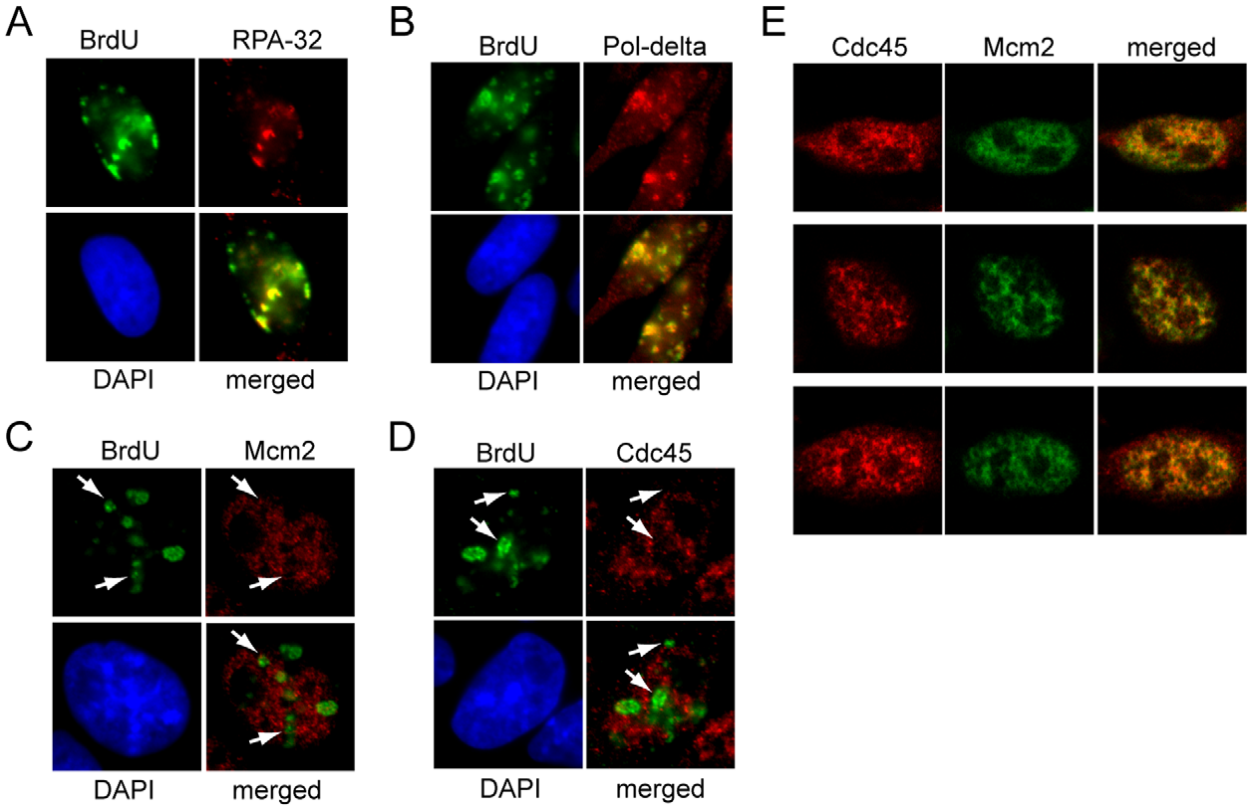
Cdc45 and Mcm2 do not noticeably colocalize with actively replicating sites *in vivo.* Asynchronous CHO cells were used in all panels. (A–D) BrdU was pulsed for 30 min and cells were fixed and stained with the indicated antibodies, and examined by IF UV-microscopy. Wide field (shown) and confocal microscopy were used and gave similar results. DAPI was used to stain nuclear DNA. Representative cells are shown. (E) Confocal microscopy was used to determine the colocalization of Cdc45 and Mcm2. Three examples are shown that are representative of the majority of cells. Chicken anti-Cdc45 (Ab2) and rabbit anti-Mcm2 (Ab3) were used in panels C–E.

RPA and DNA polymerase both colocalize significantly with replicating foci, as expected (Fig. 5A&B). In contrast, Mcm2 (chromatin-bound and non-bound) does not colocalize with replicating sites and even appears excluded from such sites (Fig. 5C, note arrowheads showing replication foci clearly lacking Mcm2), consistent with previous studies [50]. Similarly, Cdc45 (chromatin-bound and non-bound) does not colocalize with replication foci and seems likewise excluded (Fig. 5D, note arrowheads). Although only one representative cell is shown in Fig. 5D, multiple fields each containing hundreds of cells showed similar results, and other stages of S-phase nuclei showed similar findings (data not shown). However, in the majority of cells, Cdc45 does colocalize significantly with Mcm2 (Fig. 5E), as predicted for an MCM cofactor. The above experiments all utilized Cdc45 Ab1 (made in rabbit), but very similar results were obtained using Cdc45 Ab2 (made in chicken; data not shown). We conclude from these findings that although Cdc45 is rate limiting for replication foci usage (Fig. 4), it is not noticeably localized to replicating sites. Thus, Cdc45 and Mcm2 are required for DNA replication to occur, but (as discussed below) may provide a necessary function at some distance from the sites where DNA replication is actually occurring.

## Discussion

### Embryonic versus somatic replication dynamics

Sperm chromatin in *Xenopus* embryonic extracts is replicated very efficiently over an ∼1 hr duration from small 10-15-kb replicons. The preRC components are abundant in the extracts, allowing assembly of a preRC at an ORC within each 10–15-kb replicon. As described above, such preRCs contain 20–50 MCM hexamers that attract 1–2 Cdc45 molecules during initiation of replication. This frequency of preRCs and excess MCM targets for Cdc45 contribute to the efficiency and small replicon size in the embryonic system. Although there have been exceptions identified and research into mammalian origin dynamics is constantly evolving, the initiation of DNA replication in somatic cells is often very inefficient, with origins firing in some cases only once in 3–4 cell cycles [29], [55], [56]. In addition, somatic origins often correspond to broad zones of potential sites, any one of which is used extremely rarely [27], [55], [57], [58], [59], [60], [61], and replicons in somatic cells appear to be, on average, ∼10-fold larger than embryonic replicons, varying in size from 50- to 500-kb or more [23], [24], [25], [26], [27], [28], [61]. The larger nature of somatic replicons (when averaged) correlates with the fact that somatic cells require ∼10-fold longer to duplicate their genetic content relative to the embryonic system. We demonstrate here that, as for the embryonic system, the availability and dynamics of preRC components provide molecular explanations for these biological characteristics of somatic DNA replication.

### Somatic preRC stoichiometry, density, and replicon size

**Figure 6:**
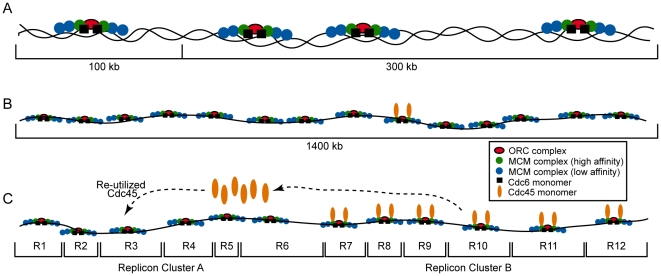
Models for preRC architecture, distribution, and usage in somatic cells. *(A)* Each 100-kb of somatic genomic DNA has, on average, one preRC comprised of one ORC hexamer, ∼4–5 total MCM hexamers (six shown for each preRC), and two Cdc6. Potentially larger replicons of 300-kb average ∼3 preRCs. *(B)* Cdc45 is extremely limiting, suggesting that only 1 in 14 preRCs/1400-kb, on average, can contain two chromatin-bound Cdc45's at any moment in S-phase. *(C)* Replicons fire in clusters and in a staggered manner. The very low, stable, and rate-limiting Cdc45 levels require that replicon cluster A wait until Cdc45 becomes available from previously-replicated clusters. Each replicon in *C* is shown with only one preRC for simplicity.

Although it is important to note that our results were obtained primarily using CHO cells as the model system, with additional tests on human HeLa and Wilson cells and mouse fibroblasts, the collective findings presented herein strongly suggest that mammalian somatic cells contain approximately 1 ORC hexamer, ∼2 Cdc6, and ∼4–5 total MCM hexamers, on average, for each 100-kb (modeled in Figure 6A). Thus, preRC density (derived from ORC levels) in somatic cells is ∼10-fold less than the embryonic situation. This offers one explanation for why somatic replicons are ∼10-fold larger and S-phase is ∼10-fold longer than in embryonic extracts. Interestingly, each 300-kb region, representative of the size of larger replicons, would average ∼3 preRCs (Fig. 6A, right side). Although preRC density may vary throughout the genome to account for smaller replicons, this also suggests that somatic cells may contain unused, dormant preRCs (both concepts discussed below). Consistent with the latter, yeast preRCs form at inactive sites [22], [62], and there are more predicted ORC/preRC sites than functional origins, with ∼one in five origins being activated to replicate the yeast genome [63], [64]. Further, embryonic chromatin contains extra preRCs by virtue of their excess of MCM hexamers, some being used in initiation, while others are dormant and used under stress conditions [20].

Another influence on somatic replicon size is the ratio of Cdc45 to preRCs. Studies in yeast and embryonic extracts have indicated that Cdc45 likely acts stoichiometrically for MCM functionality at forks, with 1–2 Cdc45 used per bidirectional fork pair [9], [10], [12], [17]. There is enough Cdc45 in embryonic extracts to provide 1–2 Cdc45 per preRC, producing 10–15-kb average distances between functional origins/preRCs. However, somatic cells have ∼2 Cdc45 available for every ∼600-kb, and only ∼2 chromatin-bound/∼1400-kb. This equates to ∼2 Cdc45 per ∼6 preRCs, or ∼2 chromatin-bound Cdc45 per ∼14 preRCs (latter is modeled in Fig. 6B). As such, the average somatic inter-origin distance *(i.e.*, replicon size) is considerably larger than the embryonic situation.

### Reconciling the presence of smaller replicons in somatic cells

Mammalian replicons can be as small as 50-kb, and under replicative stress conditions inter-origin distances can be as little as ∼15–20-kb [23], [25], [27]. Such findings suggest that preRC density should be one for each ∼50-kb, and perhaps as often as 20-kb. There are at least two explanations that can reconcile these findings. First, there is no formal evidence that all members of the preRC are located adjacent to one another in a discrete complex. Indeed, one study has shown that mammalian MCMs are not located on the same DNA fragment as ORC [53]. As such, the 4–5 total MCM hexamers/100-kb may not reside at ORC sites, which suggests an intriguing model. If MCMs are initially loaded at ORC sites, but for unknown reasons finally reside at multiple distinct sites throughout the 100-kb region, then one MCM hexamer would reside on somatic chromatin each ∼20-kb. In this situation, it would not be a complete preRC, but rather an MCM hexamer capable of unwinding DNA, every 20-kb. An alternative explanation derives from the fact that we have only determined the *average* preRC density to be one/100-kb. It may be that in some regions of the genome preRC density is one/20-kb, while in other regions it is one/400–500-kb. Indeed, conditions such as chromatin structure or transcription likely influence where preRCs can form (or not form). Thus, depending on how one models preRC distribution and stoichiometry from the approximate averages determined herein, seemingly opposing findings can be reconciled.

### Somatic origin firing is often inefficient relative to embryonic metazoan origins

Embryonic preRCs are spaced ∼10-kb and contain 20–50 MCM hexamers that attract ∼2 Cdc45. Interestingly, a similar ratio of Cdc45:MCM hexamers exists in somatic cells where ∼24–30 MCM hexamers are present for each ∼2 Cdc45 molecules. Such a ratio is essentially the same even when considering the chromatin-bound complement of somatic Cdc45 and MCMs. Thus, the ratio of Cdc45 to MCM hexamers has been maintained over developmental time. However, this physical presence of Cdc45:MCMs in somatic cells is spread over ∼600-kb. The same 600-kb span of the embryonic genome contains ∼120 Cdc45 targeting ∼1200–3000 MCM hexamers. Thus, the abundant embryonic Cdc45 protein is presented with a high density of MCM targets, whereas somatic Cdc45 is presented a significantly lower density of MCM targets on a per genomic region basis. Because of this, the ability of any *specific* somatic preRC (with an average of 4–5 MCMs) to be recognized on a genome-wide basis by low levels of Cdc45 is severely reduced relative to embryonic conditions, resulting in a decreased efficiency of preRC/origin usage in somatic cells. In agreement with this, the efficiency of Cdc45 recruitment to yeast origins/MCMs correlates directly with their firing efficiencies [22]. In addition, a recent study has shown that *S. pombe* yeast Cdc7 is limiting for initiation of DNA replication, indicating that other preRC (or pre-IC) subunits may be limiting in mammalian cells beyond Cdc45 [65].

Further compounding the above situation, mammalian ORC exhibits very little DNA-binding specificity [66]. As such, Cdc45 is presented with a low density of apparent ‘moving targets’ in terms of where preRCs/MCMs are established by ORC. This further reduces the chances (and efficiency) of any particular DNA site within a larger region from being utilized in any one S-phase, and may comprise part of the reason why mammalian origins are often composed of zones of distributed initiation sites [57], [58], [59], [67]. It is also important to keep in mind that origin efficiency in somatic cells is likely to be governed by issues beyond preRC stoichiometry. For example, complex chromatin structure (*e.g.*, heterochromatin) and transcriptional domains, both of which are of limited or no influence to embryonic chromatin, may influence origin firing in somatic cells. In addition, small intergenic regions, such as that comprising the lamin B2 origin locus, may increase the efficiency of origin usage by focusing preRC subunits into a smaller region (*i.e.*, increasing preRC/MCM density), resulting in an enhanced ability of Cdc45 to find such complexes/MCMs. Again, the preRC stoichiometries presented herein only provide ‘building blocks’ for modeling preRC structure and origin dynamics that must be factored into the larger chromatin and transcriptional environment within cells.

### Cdc45 is rate-limiting for replicon usage in somatic cells

Relative to other preRC components, the extremely low level of stable Cdc45 present at any moment in S-phase suggested that Cdc45 might be rate-limiting for replicon/preRC activation. Indeed, reduction of Cdc45 in embryonic extracts reduces DNA replication, consistent with it being rate-limiting [17]. However, if Cdc45 acts enzymatically, and not stoichiometrically, then even low levels of Cdc45 might not be rate-limiting, and its removal would also hinder replication efficiency. We show conclusively that somatic Cdc45 is indeed rate-limiting for replicon usage, and by derivation preRC activation. Introduction of additional Cdc45 molecules via microinjection causes an ∼3-fold increase in the number of active replication foci and an ∼6-fold increase in the overall level of DNA replication within nuclei. This ratio of additional foci to replication activity is consistent with the injected Cdc45 activating 3-fold the number of preRCs, and thus producing 6-fold the number of forks, that would otherwise have been active. Such a finding also supports the contention described above that the ratio of Cdc45 to MCMs influences the efficiency of preRC/origin usage. Intriguingly, although we can artificially introduce additional Cdc45 during S-phase itself, followed by acute changes to replicon/preRC activation, our colony forming assays indicate that mammalian cells do not tolerate permanent changes to Cdc45 expression during cell cycling.

Our results are in close agreement with those shown in fission yeast where overproducing Cdc45 causes an increase in DNA replication activity at a number of origins [22], indicating that Cdc45 is rate-limiting for DNA replication in that system. Although the relative abundance of Cdc45:MCM subunits is not known in such yeast, the authors proposed that Cdc45 is therefore likely present at low (*i.e.*, limiting) levels in cells, which prevents efficient origin usage. We show here that in somatic cells both situations are true. Cdc45 is stable and present at low levels that are limiting to replicon/preRC activation at any moment in S-phase. Additional replicons/preRCs can be acutely activated upon introduction of more Cdc45, indicating that Cdc45 is minimally one factor that established preRCs wait for in order to become activated. However, as described above, chromatin accessibility, transcriptional domains, or other as yet unknown limiting factors may also control replicon usage.

### Replicon clusters and staggering

DNA replication in *Xenopus* extracts and in mammalian cells occurs in replicon clusters, with clusters replicating in a staggered manner [20], [23], [25]. In somatic mammalian cells, the low level of stable and rate-limiting Cdc45 molecules present throughout S-phase provides a molecular explanation. If early-firing replicon clusters are occupied by stoichiometrically required Cdc45 (*e.g.*, cluster B in Fig. 6C), then there is not enough Cdc45 for other clusters to be simultaneously occupied (*e.g.*, cluster A). Such later-firing replicon clusters will remain inactive until Cdc45 becomes available from the limited, stable pool of Cdc45 acting at earlier clusters. Thus, complete replicon usage during S-phase can be achieved only by re-utilizing the small number of rate-limiting Cdc45 molecules, which staggers replicon usage. We propose a ‘function-and-run’ mechanism where Cdc45 leaves completed replicon clusters, perhaps from forks that have merged (while those at the cluster periphery continue elongating), and migrates to later-firing clusters/replicons (Fig. 6C). Consistent with this model, Cdc45 can re-enter pre-established forks/preRCs during S-phase in both yeast and *Xenopus* [9], [12].

### Reconciling the low level of MCMs with the existence of excess, dormant MCMs

As in the embryonic system, mammalian somatic cells contain an excess of MCMs that are dormant during S-phase, but become functionally required under replicative stress [20], [25], [68]. Total levels of somatic MCMs can be reduced by perhaps ∼75% with no adverse effect on DNA replication [25]. This indicates that excess MCMs exist in somatic cells and appears to be inconsistent with our results showing that MCMs are present at low levels relative to the embryonic situation. However, these findings can be reconciled if one considers the effect on preRC architecture after a reduction of somatic MCMs as reported by others.

Reducing the 4–5 MCM hexamers/ORC that we estimated by ∼75% produces ∼1–2 MCMs/ORC. In *Xenopus* extracts, 2 MCM hexamers/ORC are minimally required for DNA replication [18], and our estimations suggest that ∼2 out of the 4–5 total MCM hexamers may be actively utilized (*i.e.*, higher affinity for chromatin) at any one moment in S-phase relative to each ORC (green circles for higher affinity MCMs in Fig. 6). Therefore, it seems likely that a somatic preRC could function with ∼75% of its MCM complement absent. Further, if a larger replicon of 200–300-kb contains multiple preRCs (*e.g.*, 2–3 preRCs as described above), and only one preRC is required for activation, then even more MCMs would be present and could be removed without an adverse effect on the replicon. Indeed, a recent study has shown that mammalian cells can proliferate with ∼90% of their MCM complement reduced [68]. However, such cells cease dividing in a few generations and accumulate DNA damage, and our results would indicate that this is because a threshold has been reached where preRCs have been reduced to less than 2 available MCM hexamers/ORC. Thus, from another perspective, somatic MCMs can be considered in excess, but just not as excessive in total numbers per genome as that in embryonic systems. Intriguingly, if somatic replicons do indeed contain ∼2–3-fold the preRCs/MCMs they need for functionality, then this may explain why our Cdc45 microinjection experiments produce an ∼3-fold increase in foci/preRC activation, but no increase beyond this even though we have provided ∼10-fold excess of Cdc45/nucleus.

### Paradoxical lack of Cdc45 in replication foci

There is a longstanding paradox in mammalian somatic cells that, although presumably required for helicase functionality at forks during DNA replication (*i.e.*, within replisomes), MCMs are not localized by immunofluorescent staining techniques to actively replicating foci (*i.e.*, BrdU incorporation sites), but are instead clearly present at distal DNA sites throughout the genome that have not yet replicated [50], [51], [52], [53], [54]. We now show that in addition to MCMs, Cdc45 is also not noticeably present at actively replicating sites *in vivo*. We do find that Cdc45 localizes to at least a subset of Mcm2 sites, consistent with its presumptive role as an MCM cofactor [9], [69]. RPA, PCNA, and DNA polymerase delta are clearly present in replicating foci [ref. [50] and results herein], indicative of the presence of ssDNA at the forks in the foci. Given the clear presence of such fork proteins in replication foci, the lack of clearly visible MCMs and Cdc45 within replicating foci creates a paradox in which MCM-Cdc45 complexes facilitate DNA unwinding while not seemingly localized directly at forks within replication foci. In addition, MCM-Cdc45 are known to interact with DNA polymerases via Mcm10 [70], [71], which should place them within replication foci (replisomes). We suggest some possibilities below that may reconcile these paradoxical findings.

We concede that a limitation of our immunofluorescence assays may be that the absence of Cdc45 at replication foci derives from a lack of Cdc45 epitopes in the foci, but this would need to be true for both of the polyclonal antibodies we tested (and similarly true for the multiple MCM studies described above that arrive at the same conclusion). Also, the timing of fixation relative to when/where DNA replication is occurring might contribute to a seeming lack of colocalization, although this does not affect colocalization of RPA, PCNA, or DNA polymerase with BrdU foci. However, a possible explanation for this apparent distal localization paradox may be that MCM-Cdc45 complexes do indeed function directly at forks in the foci, but also simultaneously associate with and provide an unknown function during DNA replication at distal DNA sites (see below). Alternatively, MCM-Cdc45 complexes might actually function at a significant distance, in terms of DNA length, from replication forks themselves, but are brought into replisome proximity during DNA replication via a coordinated looping process, perhaps anchored to replisomes by Mcm10 [70], [71]. In either situation, it is entirely possible that large-scale chromosomal rearrangements during fixation steps for IF imaging and BrdU analysis, as can occur [72], might cause an artificial dissociation of Cdc45/Mcm2 from replication foci due to such distal DNA interactions, leading to the observation that MCM-Cdc45 complexes are not physically present in replication foci.

It is well known that *in vitro* dsDNA strand separation assays indicate that MCMs have the potential to function directly as a helicase [73], [74], and at least one report has suggested that Cdc45 and MCMs are present at replication forks in metazoan embryonic extract replication assays [69]. The latter study utilized a streptavidin steric hindrance method to accumulate active replication forks at one site on a plasmid, along with accumulation of proteins present in replisomes at such forks. Cdc45 and MCMs were enriched at these accumulated forks, suggestive of their functional presence within active forks [69]. However, an alternate interpretation of this study is that Cdc45 and MCMs might have been initially functioning at distal sites (*e.g.*, at the streptavidin site itself) from where replisomes were functioning on the plasmid, and the streptavidin caused replisomes to accumulate at this site, which then overlapped some of the Cdc45 and MCM complexes (*i.e.*, they caught up with one another). Consistent with this alternate interpretation, MCMs are indeed known to interact with a significant portion of plasmid DNA sequences in *Xenopus laevis* embryonic extracts [75].

It must also be kept in perspective that the *in vitro* helicase assays used to demonstrate the ability of purified MCMs to separate dsDNA oligonucleotides [73], [74] may not be a true indicator of MCM function *in vivo*. Although MCMs do indeed separate these structures (*i.e.*, act as helicase), as carefully discussed in a recent report by the Diffley lab [76], the RuvB protein involved in recombination, and which does not actually function as a helicase, also separates dsDNA strands in this helicase assay as a result of its translocase activity. Thus, this *in vitro* helicase assay itself, while consistent with a role for MCMs in direct fork functionality, does not conclusively validate that MCMs do indeed function as such under normal biological conditions. However, such assays clearly indicate that MCMs possess translocase activity, recently confirmed using other approaches [76]. Intriguingly, the latter study also showed that yeast MCMs assembled on circular plasmid DNA do not possess an intrinsic ability to melt dsDNA, but do translocate on DNA molecules [76]. Despite these caveats, if a fixation artifact is indeed concealing the fact that mammalian MCM-Cdc45 is localized at forks during DNA replication, then it becomes much easier to reconcile these findings. However, if MCM-Cdc45 complexes do function exclusively at DNA sites that are separated a significant distance from forks themselves, whether or not they are brought into physical proximity within replisomes during replication (via Mcm10 interactions/looping), then how might their functionality at a distance facilitate DNA replication?

We favor two possible explanations that are not mutually exclusive with one another, and that incorporate the translocase activity of MCMs. First, as suggested by Laskey and Madine [54], MCM-Cdc45 complexes may function as a fixed translocase at distal sites, producing DNA unwinding within replication foci due to rotation and resultant torque forces applied to the DNA helix at a location far from the fork/ssDNA itself (rotary pump model). However, this should not be misinterpreted to imply that ssDNA is created the entire distance between distal MCMs and the forks. Second, we propose that MCM-Cdc45 might also facilitate fork unwinding from a distance by translocating along DNA (or the DNA moves through fixed MCM translocases, to be consistent with the rotary pump model) and altering higher order chromatin structure indirectly with associated chromatin modifying enzymes. In such a situation, simpler less condensed chromatin (*e.g.*, ‘beads on a string’) would be presented to the replisomes in a coordinated manner. Such decondensation of chromatin structure may secondarily be accompanied by changes to DNA structure/writhing, resulting in a more unwound DNA topological state at distal forks within replication foci. Indeed, MCMs interact strongly with histone H3 [77], [78], and with chromatin modifying enzymes [77]. In addition, we have shown that Cdc45 promotes DNA replication in part via changes to higher order chromatin decondensation [37], which could occur at distal sites from forks. Either possible mechanism might thus produce "indirect helicase functionality at a distance", and perhaps provide other functionality. Clearly, further research is necessary to explain molecularly the paradoxes observed for MCMs/Cdc45 in mammalian somatic cells.

## Materials and Methods

### Cell Culture, synchronization, and transfections

Chinese hamster ovary (CHO) [39], Wilson human Burkitt's lymphoma line [79], NIH3T3, HeLa, and SW480 colon carcinoma cells (latter three cell lines available from ATCC) were maintained in MEM with 10% Fetal Clone II (Hyclone). CHO cells were arrested in G_0_ by isoleucine starvation [39]. Transfections used Fugene-6 (Roche) and equal amounts of plasmids expressing indicated cDNAs in pcDNA3-based vectors co-transfected with equal amounts of pTK-hygro. Colony selection in hygromycin (400 µg/ml) lasted 10 days.

### Protein purification

The following were expressed with His6-tags in BL21-CodonPlus-DE3-RIPL bacteria (Stratagene): CgOrc2, CgOrc4, CgOrc5, HsMcm2, HsMcm4, HsMcm5, HsMcm6, HsMcm7, CgCdc6, and CgCdc45 (Cg, *Cricetulus griseus*, Chinese hamster). Except for HsMcm2 and CgCdc6, proteins were largely insoluble and required purification under denaturing conditions using Talon columns (Clontech). Non-tagged CgCdc6 was also purified over HiTrap Sp FF and HiTrap Heparin HP columns (details available). Purified proteins were separated in gels alongside ultrapure BSA standards (Biorad), and coomassie-stained bands were compared to determine the amount of each protein isolated.

### Antibody production

Polyclonal rabbit antibodies were produced by Covance, Inc., and polyclonal chicken antibodies (where indicated) were produced by Aves Labs. All were used at 1∶500 unless specified. Anti-CgOrc2 (Ab3; 1∶100 for Westerns), anti-Mcm2 (Ab3; 1∶50 for IF), anti-HsMcm4 (Ab1), anti-HsMcm5 (Ab2), anti-CgCdc6 (Ab2), anti-CgCdc45 (Ab1), chicken anti-HsMcm4 (Ab2), and chicken anti-CgCdc45 (Ab2; 1∶50 for IF). Purified, antigen-specific antibodies: whole antisera were incubated with full-length antigens immobilized on blotting strips, then eluted [80].

### Additional antibodies

All antibodies were generated in rabbits and used at 1∶500 unless specified. From BD Biosciences: mouse monoclonal anti-HsOrc4 (#611171; conserved domain for Hs and Cg), anti-HsOrc5 (#559267; identical C-term epitope except for one residue between Hs and Cg), and anti-HsMcm2 (Ab1; #559542). Anti-HsOrc2 (Ab1), anti-HsOrc2 (Ab2), anti-HsMcm2 (Ab2), anti-HsMcm5 (Ab1), and anti-HsMcm7 (Ab1) were provided by Rolf Knippers (Konstanz, Germany). From Santa Cruz: anti-HsCdc6 (Ab1; sc#8341), mouse anti-Mcm7 (Ab2; sc#9966), and goat anti-polymerase delta (1:50). From Upstate: anti-Hs-cyclin A (1 µg/ml). From Calbiochem: mouse anti-lamin A/C, anti-tubulin, and anti-HsPCNA (1.5 µg/ml). Anti-RPA-32 rat polyclonal was from Cell Signalling (1∶100).

### Protein quantitation and fractionation assays

Cells were scraped off plates into cold PBS (without trypsinization to prevent protein loss), then lysed and boiled immediately in loading dye. Prior to such lysis, an aliquot of scraped cells was removed to determine the total number of cells collected. The aliquot of cells was resuspended in 10 mM HEPES, 10 mM EDTA, on ice for 15 min to yield single-cell suspensions without using trypsin. Cell numbers were determined with several hemacytometer readings of multiple fields, done independently by two individuals (all within 5%). Cells were collected on different days to generate two independent lysates for quantification. For an antibody used two times to quantify, each assay was done on independent lysates. Immunoblotting and immunoprecipitation were performed using standard techniques. Fractionation into soluble and chromatin lysates was done as described [39].

### Quantitative mass spectrometry

Protein bands were excised from gels, dehydrated, reduced with tris(2-carboxyethyl)phosphine, alkylated with iodoacetamide, and digested overnight with trypsin. Resultant peptides were extracted, concentrated, and selected for multiple reaction monitoring experiments using a nanoflow liquid chromatograph (U3000, Dionex, Sunnyvale, CA) coupled to a triple quadrupole mass spectrometer (TSQ Quantum Ultra, Thermo, San Jose, CA). A synthetic peptide was spiked into each sample prior to QMS. Peptides were separated at 300 nl/min by reverse phase chromatography (LC Packings C18 Pepmap, 75 um ID×15 cm) using a 25 minute gradient from 5% A to 50% B (A: 2% acetonitrile/0.1% formic acid; B: 90% acetonitrile/0.1% formic acid). Three sequence-specific transitions (y- ions) were monitored for each peptide.

### Microinjections and computational analyses

Soluble His6-CgCdc45 was purified from bacteria and diluted in PBS containing 100 µM Cy3-dUTP (Amersham). The control was from bacteria transformed with His6-vector (both samples contained residual bacterial proteins). The final concentration of Cdc45 injected into CHO nuclei was 45 ng/µl. An Eppendorf FemptoJet and InjectMan instrument was used on a Nikon Eclipse TE2000-S microscope outfitted with a QImaging Retiga 1300 CCD camera and a LWD 20x/0.40 NA lens. Injections used 300 hPa for 0.4 sec, resulting in ∼1 picoliter injected/nucleus. Thus, ∼45 fg of Cdc45 was injected/nucleus, which is ∼10-fold the existing Cdc45 per CHO cell (∼3 fg/nucleus based on Table S2). Equal numbers of CHO nuclei were injected and fixed 30 min later with 2% formaldehyde. Unincorporated Cy3-dUTP was washed away. Images of injected cells were obtained using a Leica TCS SP5 AOBS confocal microscope and LAS AF software (ver 2.1.0), with no file compression. All settings were identical during image acquisition for valid intensity quantification comparisons. Images of individual nuclei were obtained for clarity using 2.5X optical zoom. For foci counting, the gain was reduced on Cdc45-injected nuclei prior to separate image acquisition. Intensity and foci analysis was done using Image Pro Plus (ver 6.2) (Media Cybernetics, Inc.). Identical threshold settings and measurements were used to generate the mean intensity and foci data. Foci were defined as an object with an intensity value of at least 100 (dynamic range) and an area of between 10–600 pixels. Each nucleus was examined independently. No additional post-processing manipulations were done to enhance images.

### Immunofluorescence

Standard methods were used to process cells for indirect immunofluorescence and BrdU labeling [81]. Wide field microscopy utilized a Zeiss Automated Upright Fluorescent microscope, and confocal images were collected using a Leica TCS SP5 Laser Scanning microscope. Adobe Photoshop software was used to process and arrange images.

### Formula, molecular weights, and genome sizes

The formula used to quantify the number of protein molecules was: (ng of unknown protein)×6.02×10^23^/(molar weight of protein in ng)×(number of cells analyzed), which represents the number of molecules of each protein per cell. MWs were used based on the atomic mass of amino acids in each protein (in Daltons): Orc2 = 65,700; Orc4 = 50,400; Mcm2 = 102,000; Mcm4 = 96,600; Mcm5 = 82,300; Mcm7 = 81,300; Cdc6 = 62,300; Cdc45 = 65,400. Although Orc5 is predicted to be 50,300 Da, the apparent sizes of two Orc5 isoforms were used: ∼45 and 60 kDa; also seen by others [32]. The Chinese hamster diploid genome is ∼7×10^9^ bp (http://www.cbs.dtu.dk/databases/DOGS), and was used for calculations. Mouse (∼6.8×10^9^ bp) and human (∼6.8×10^9^ bp) diploid genomes are similar. There are ∼70,000 100-kb DNA segments per diploid mammalian somatic cell genome.

## Supporting Information

Figure S1 **Characterization of antibodies.** Total cellular extracts (*TCE*), chromatin-enriched (*Nucl*), or total extracts from cells transiently-transfected with LacI-tagged subunits were used. Expected sizes for endogenous ORC, MCM, Cdc6, or Cdc45 are indicated with MW. Expected sizes of LacI-tagged versions are also indicated. For MCMs, the lower arrow is the endogenous, and upper arrow is the LacI-tagged version. The anti-Mcm7 also recognizes LacI-HsMcm7 (not shown). In *C*, An IP-Western verified Cdc6-Ab1 and Cdc6-Ab2 specificities. Anti-Cdc6 Ab2 recognizes three protein bands, arbitrarily assigned p60, p62, and p64 (likely due to ubiquitinylation and/or phosphorylation as described in the text). LacI-CgCdc6 is p100. Transfection experiments using shRNA constructs directed at CgCdc45 and CgCdc6 were undertaken in CHO cells to determine which exact bands corresponded to Cdc45p and Cdc6p, respectively, but were inconclusive. For Cdc45 in particular, several days of shRNA expression failed to effectively reduce expression of Cdc45 protein bands, which we attribute at least in part to the noticeable stability of Cdc45 protein as indicated in our results in Figure 4.
(TIF)

Figure S2 **Protein estimations for hamster, human, and mouse Orc2, Orc4, Orc5, Cdc6, and Cdc45.** Increasing amounts of bacterially-expressed and purified CgORC subunits (*A*), CgCdc6 (*B*), and CgCdc45 (*C*) were used to estimate subunit levels in the indicated number of asynchronous CHO, 3T3, HeLa, or Wilson cells. ng estimated are shown below each panel and refer to each protein band present. Representative immunoblots are shown.
(TIF)

Table S1 **Antibodies used in this study.** For sources: com, commercial; RK, Rolf Knippers laboratory; here, generated for this study; as, antiserum. Purified indicates that the antibodies were affinity-purified against the antigen, or for commercial derivatives are purified immunoglobulins. If a protein is listed under Immunogen without any peptide or domain specification, then full-length protein was used as the immunizing antigen. If full-length purified proteins for immunizing contained tags, then such tags are listed in the name of the protein.
(TIF)

Table S2 **Quantification results for preRC subunits in somatic Chinese hamster ovary (CHO) cells.** Superscript definitions: (a) Obtained from CHO data, +/− the estimated ng errors in reading the blots. (b) p60 and p45 Orc5 isoforms were analyzed together on the same two blots, not four blots. (c) ORC averages do not include the p45-Orc5 data. (d) The ∼percentage of each preRC subunit chromatin-bound at 9-hr in the immunoblots of Fig. 3. (e) One of each of the three blots analyzed included non-tagged CgCdc6. *ND*, not determined; *N/A*, not applicable; *NS*, not shown.
(TIF)

Table S3 **Raw data for 25 most intense, and measurable, microinjected nuclei for each condition.** Early-S (stage-1) nuclei were used for these analyses to facilitate fair and equal computations across conditions. The mean overall Cy3-dUTP intensities and mean number of foci were determined as described in Methods. Errors are +/− 1 standard deviation.
(TIF)
