# Supplementary figures and images for: Cdc45 Limits Replicon Usage from a Low Density of preRCs in Mammalian Cells
Source: PLoS One. 2011 Mar 1;6(3):e17533. doi: 10.1371/journal.pone.0017533 (PMC3046982; doi:10.1371/journal.pone.0017533)

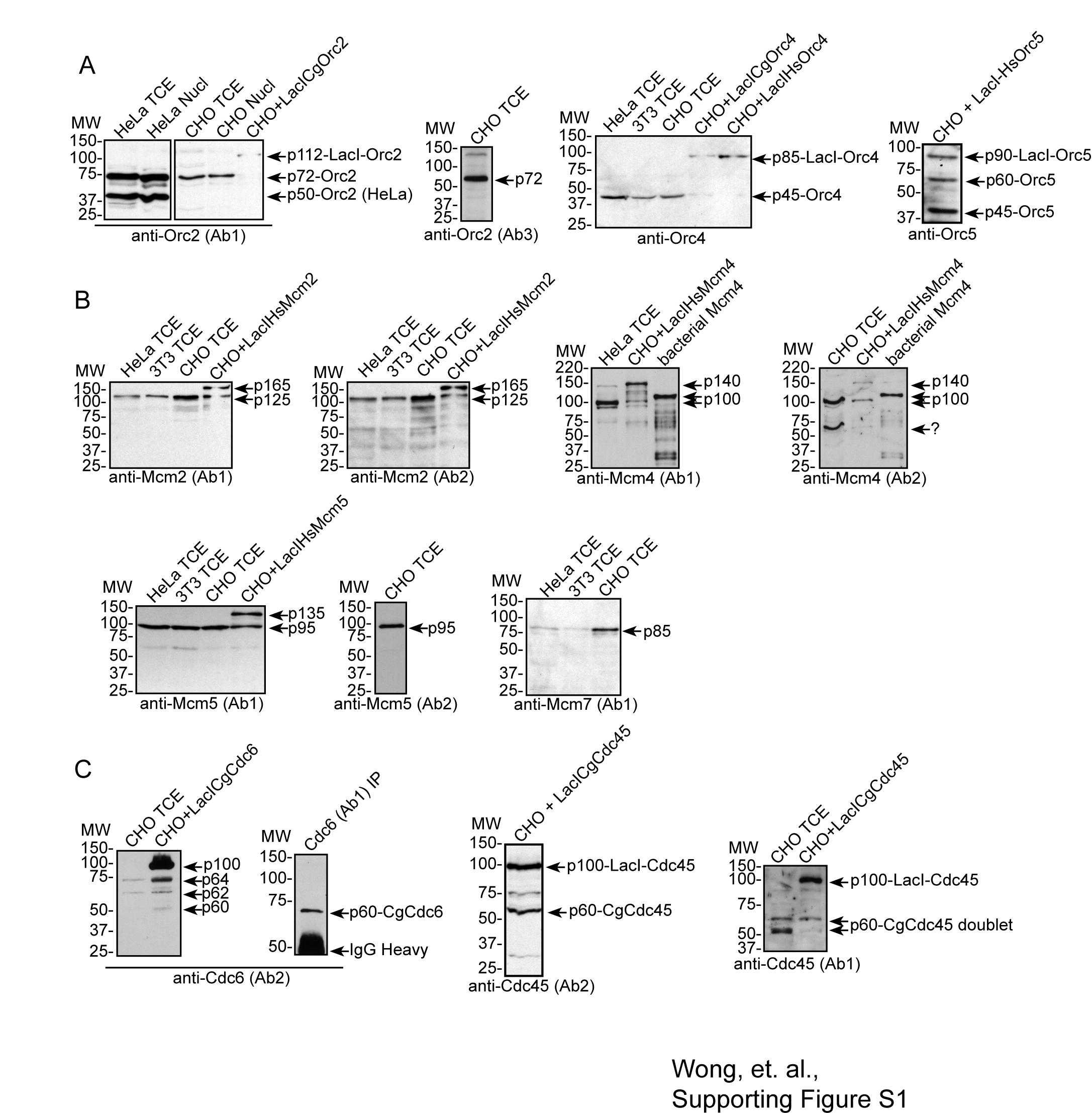

Supplement: Figure S1 — Characterization of antibodies. Total cellular extracts (TCE), chromatin-enriched (Nucl), or total extracts from cells transiently-transfected with LacI-tagged subunits were used. Expected sizes for endogenous ORC, MCM, Cdc6, or Cdc45 are indicated with MW. Expected sizes of LacI-tagged versions are also indicated. For MCMs, the lower arrow is the endogenous, and upper arrow is the LacI-tagged version. The anti-Mcm7 also recognizes LacI-HsMcm7 (not shown). In C, An IP-Western verified Cdc6-Ab1 and Cdc6-Ab2 specificities. Anti-Cdc6 Ab2 recognizes three protein bands, arbitrarily assigned p60, p62, and p64 (likely due to ubiquitinylation and/or phosphorylation as described in the text). LacI-CgCdc6 is p100. Transfection experiments using shRNA constructs directed at CgCdc45 and CgCdc6 were undertaken in CHO cells to determine which exact bands corresponded to Cdc45p and Cdc6p, respectively, but were inconclusive. For Cdc45 in particular, several days of shRNA expression failed to effectively reduce expression of Cdc45 protein bands, which we attribute at least in part to the noticeable stability of Cdc45 protein as indicated in our results in Figure 4. (TIF) [file pone.0017533.s001.tif]

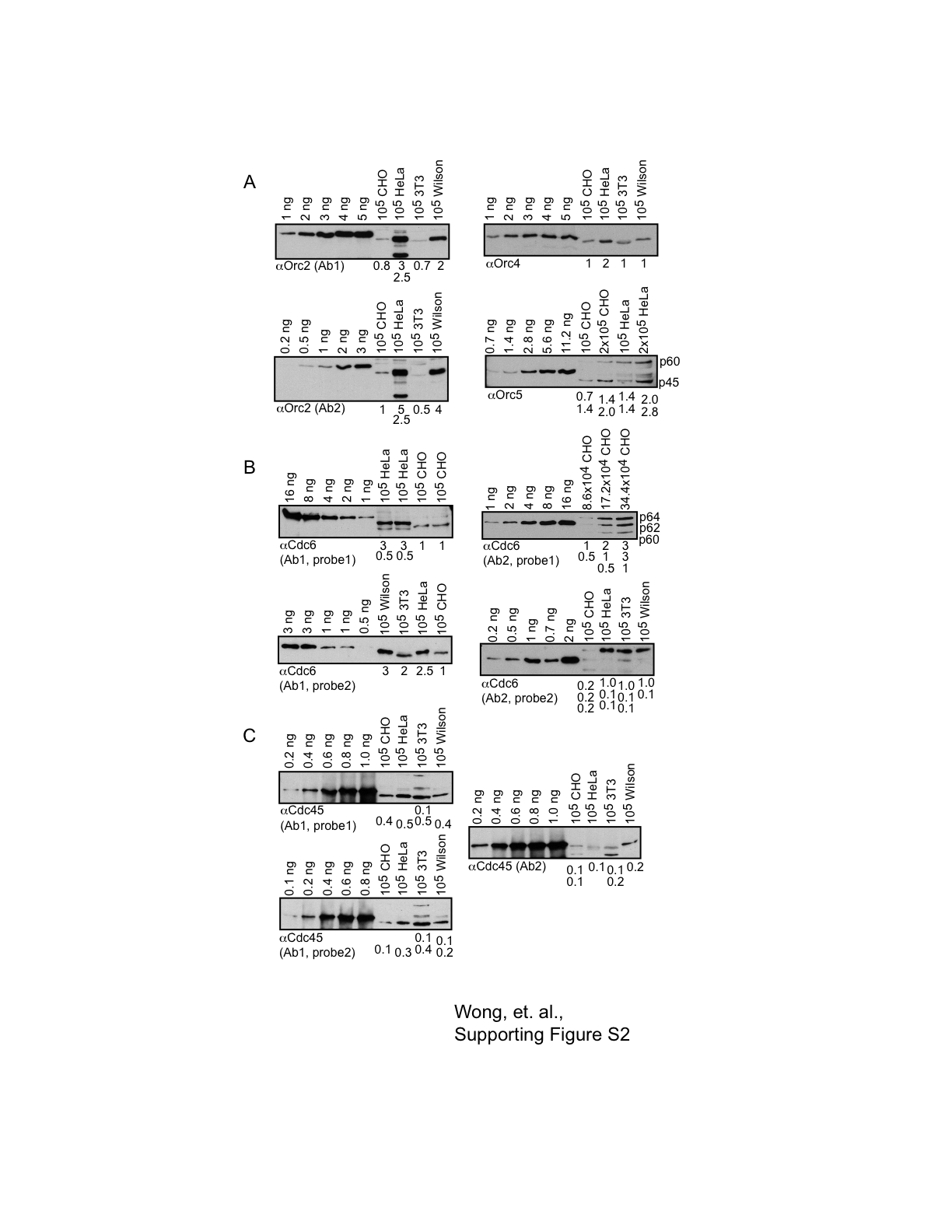

Supplement: Figure S2 — Protein estimations for hamster, human, and mouse Orc2, Orc4, Orc5, Cdc6, and Cdc45. Increasing amounts of bacterially-expressed and purified CgORC subunits (A), CgCdc6 (B), and CgCdc45 (C) were used to estimate subunit levels in the indicated number of asynchronous CHO, 3T3, HeLa, or Wilson cells. ng estimated are shown below each panel and refer to each protein band present. Representative immunoblots are shown. (TIF) [file pone.0017533.s002.tif]

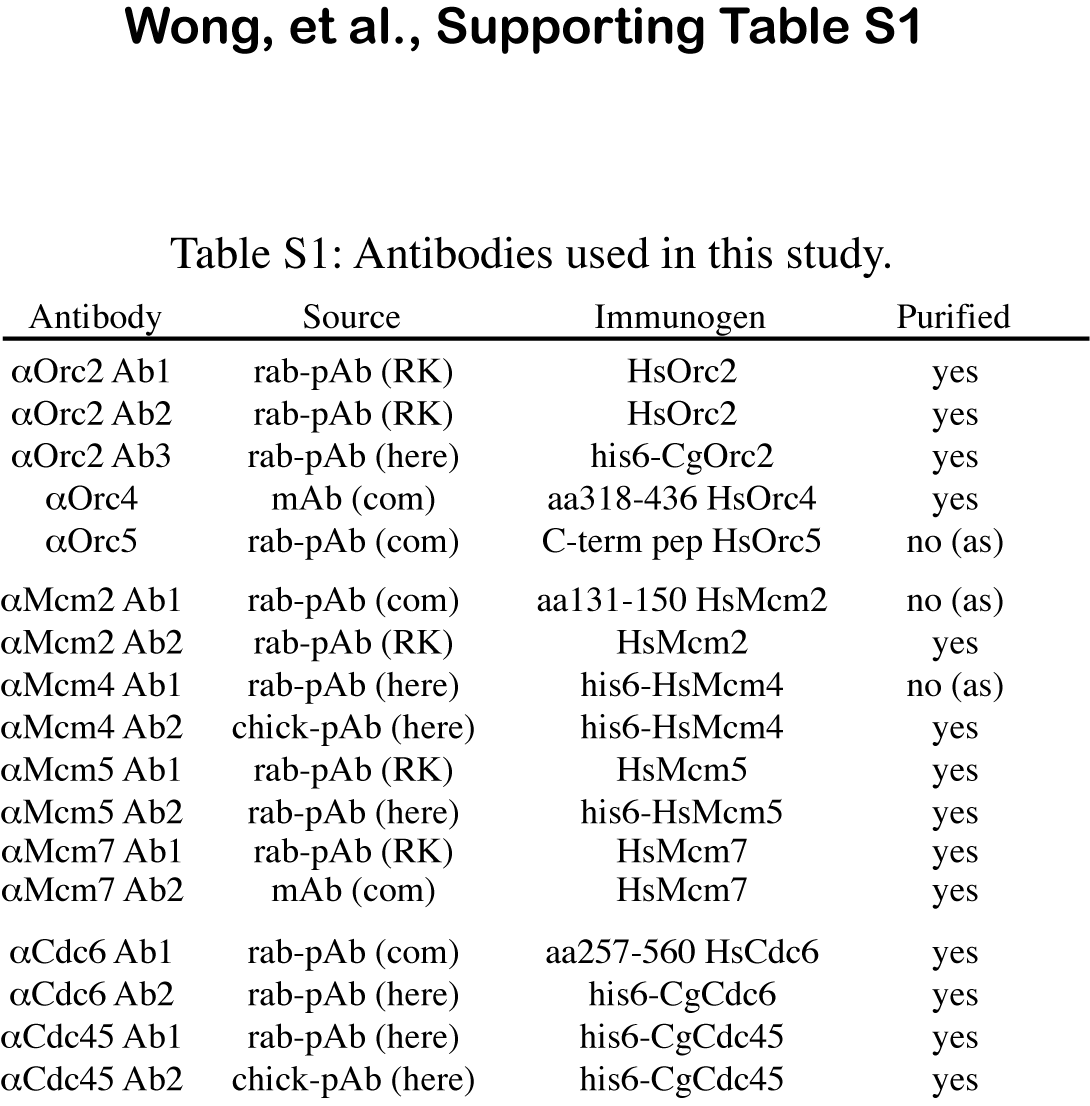

Supplement: Table S1 — Antibodies used in this study. For sources: com, commercial; RK, Rolf Knippers laboratory; here, generated for this study; as, antiserum. Purified indicates that the antibodies were affinity-purified against the antigen, or for commercial derivatives are purified immunoglobulins. If a protein is listed under Immunogen without any peptide or domain specification, then full-length protein was used as the immunizing antigen. If full-length purified proteins for immunizing contained tags, then such tags are listed in the name of the protein. (TIF) [file pone.0017533.s003.tif]

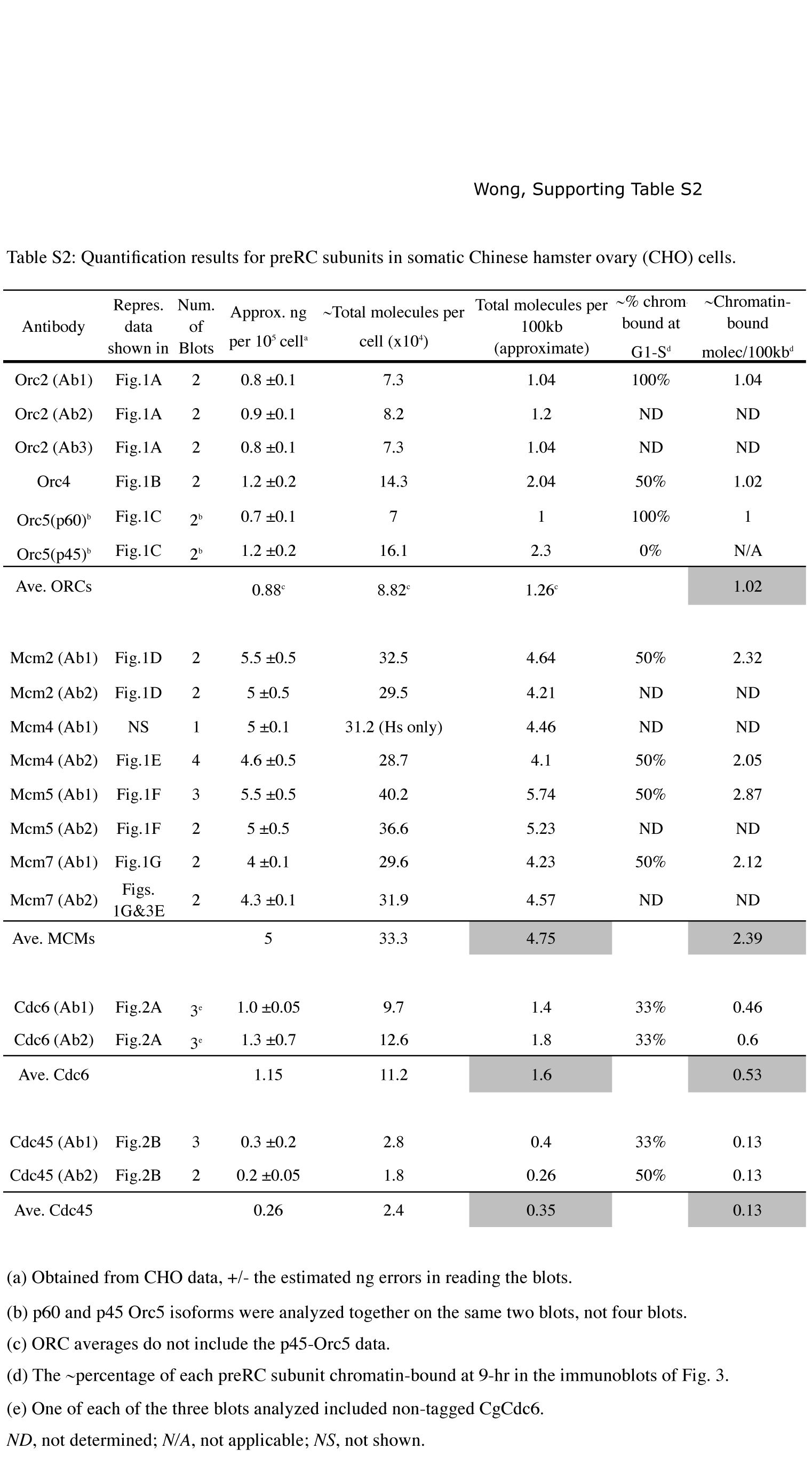

Supplement: Table S2 — Quantification results for preRC subunits in somatic Chinese hamster ovary (CHO) cells. Superscript definitions: (a) Obtained from CHO data, +/− the estimated ng errors in reading the blots. (b) p60 and p45 Orc5 isoforms were analyzed together on the same two blots, not four blots. (c) ORC averages do not include the p45-Orc5 data. (d) The ∼percentage of each preRC subunit chromatin-bound at 9-hr in the immunoblots of Fig. 3. (e) One of each of the three blots analyzed included non-tagged CgCdc6. ND, not determined; N/A, not applicable; NS, not shown. (TIF) [file pone.0017533.s004.tif]

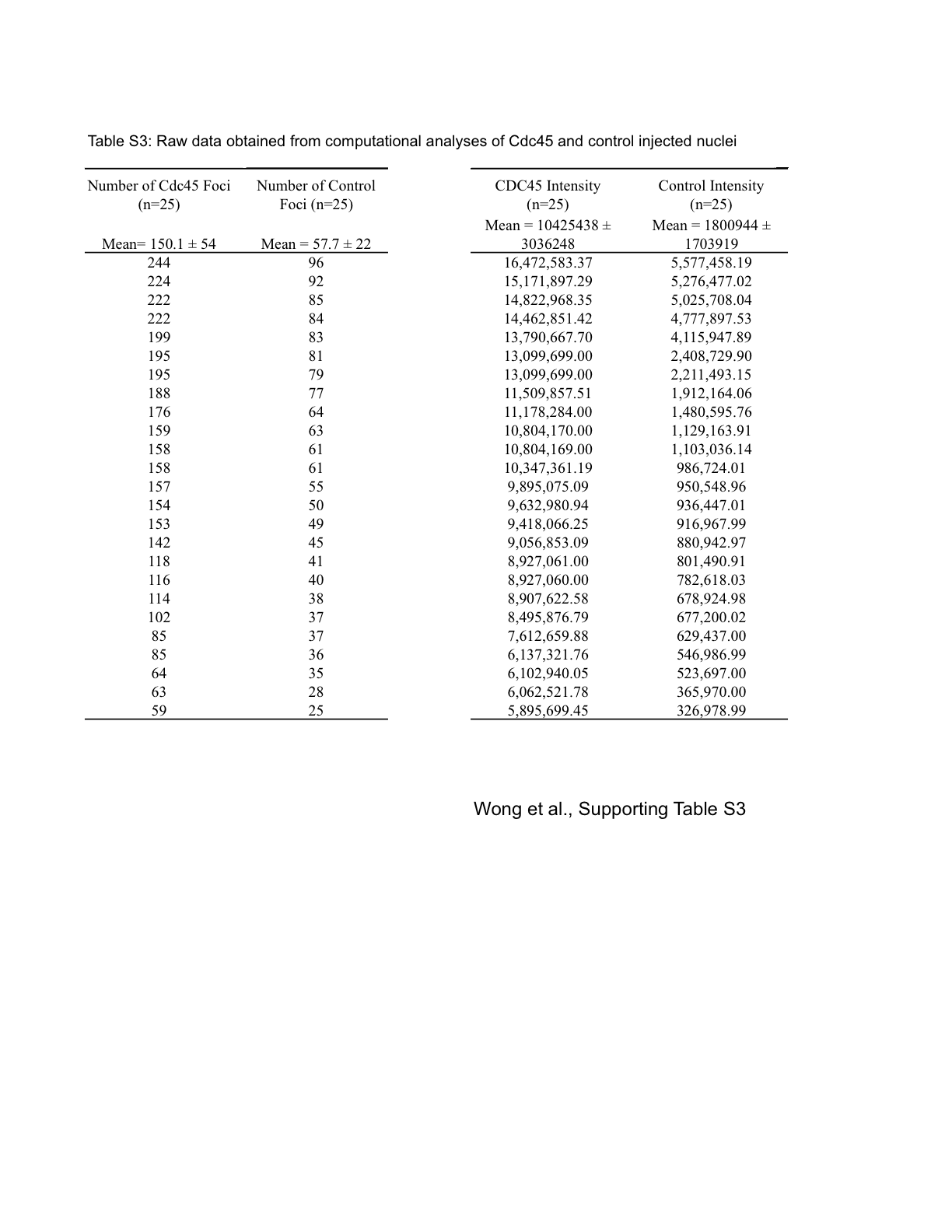

Supplement: Table S3 — Raw data for 25 most intense, and measurable, microinjected nuclei for each condition. Early-S (stage-1) nuclei were used for these analyses to facilitate fair and equal computations across conditions. The mean overall Cy3-dUTP intensities and mean number of foci were determined as described in Methods. Errors are +/− 1 standard deviation. (TIF) [file pone.0017533.s005.tif]
